# 자율신경균형 증진 간호중재를 위한 생행동적 이론적 기틀 구축: 세포노화 기전 기반으로

**DOI:** 10.7586/jkbns.24.008

**Published:** 2024-05-28

**Authors:** Nahyun Kim, Jooyeon Park

**Affiliations:** 1 College of Nursing, Keimyung University, Daegu, Korea; 1 계명대학교 간호대학; 2 College of Nursing, Daegu University, Daegu, Korea; 2 대구대학교 간호대학

**Keywords:** Psychological stress, Cellular senescence, Autonomic nervous system, Models, theoretical, 심리적 스트레스, 세포노화, 자율신경계, 이론적 기틀

## Abstract

**Purpose:**

This study reviewed the pathophysiological mechanisms of cellular aging caused by psychological stress and aimed to establish a biobehavioral theoretical framework for nursing interventions to promote autonomic balance based on these mechanisms.

**Methods:**

A comprehensive literature review was conducted.

**Results:**

A review of the literature showed that the stress response increases the secretion of catecholamines and glucocorticoids, resulting in a greater allostatic load. This load induces inflammatory reactions and oxidative stress, shortening telomere length and damaging mitochondrial DNA, which can lead to cellular aging. Based on this mechanism, a biobehavioral theoretical framework for nursing interventions was established. This framework focuses on delaying or inhibiting the cellular aging process by acting on the stress response stage and improving autonomic balance.

**Conclusion:**

According to the proposed biobehavioral theoretical framework, stress-relieving nursing interventions may act on the mechanism of cellular aging caused by stress responses. We believe that this framework could expand our understanding of the biobehavioral aspects of stress and would facilitate efforts to use biomarkers to evaluate the effectiveness of stress-related nursing interventions at the cellular level.

## 서론

### 1. 연구의 필요성

만성적 스트레스는 다양한 신체적 및 심리적 건강에 부정적인 영향을 미칠 수 있는 주요 요인이 되고 있으며, 최근에는 세포노화, 신체노화 및 노화관련질환과도 밀접한 관련이 있는 것으로 보고되면서 스트레스가 세포노화에 미치는 기전에 관한 연구가 활발하게 이루어지고 있다[1-4]. 자율신경계는 스트레스에 노출되었을 때 가장 먼저 활성화되는 생리적 기전으로, 시상하부-뇌하수체-부신 축(hypothalamic-pituitary-adrenal [HPA] axis)을 활성화하여 코티졸(cortisol)을 분비함으로써 스트레스 상황에 적절하게 대처할 수 있게 해준다[5,6]. 그러나 만성적이고 반복되는 스트레스는 심리적으로 우울, 분노, 정서불안, 긴장 등을 초래하여 심리적 병태생리(psychopathology) 기전을 촉발시키고, 생리적으로는 자율신경계를 과다하게 활성화함으로써 심혈관질환, 당뇨병, 대사증후군, 염증성질환, 비만, 뇌졸중, 암 등과 같은 다양한 노화관련질환을 초래하는 것으로 알려져 있다[6-9].

만성적 스트레스로 인한 자율신경계의 과다활성은 자율신경계의 불균형을 초래하고 그 결과 염증성 사이토카인(cytokine) 분비 및 체내 산화스트레스 수준을 증폭시켜 결국 세포노화를 촉진하게 된다[9-11]. 세포노화의 과정은 분자생물학적으로 주요 유전물질이자 영향인자인 텔로미어와 미토콘드리아 deoxyribo nucleic acid (DNA) 구조 및 기능의 변화와 관련지어 설명되고 있다[4,12]. 이에 텔로미어 길이와 미토콘드리아 DNA는 세포노화를 반영하는 주요 지표로 널리 측정되고 있으며[13,14], 다수의 연구에서 스트레스와 이들 세포노화 지표들간의 관련성에 관한 의미있는 근거를 제시해 주고 있다[3,4,9,15-17].

이처럼 만성적 스트레스로 인한 심리적, 생리적 불균형이 세포내 환경을 변화시키고 세포노화를 촉진하여 병적 상태를 초래할 수 있다는 사실이 알려지면서[18] 스트레스를 완화시키기 위한 다양한 중재들이 소개되어 왔다. 대표적으로 이완요법, 명상요법, 마음챙김요법, 운동요법, 음악요법 등이 잘 알려져 있는데, 이러한 비약물적 중재들은 대체로 교감신경 활성도를 낮추고 부교감신경 활성도를 증가시킴으로써 자율신경기능의 균형을 회복시키는데 초점을 두고 있다[19,20]. 그러나 다수의 중재연구에서 중재의 내용이 병태생리적 기전에 기반을 두기보다는 스트레스 자극에 대한 인지 변화 및 증상 완화 기법에 중점을 두거나 중재의 효과를 심리적, 정서적 측면에 초점을 두고 있어 스트레스의 생행동적 측면을 충분히 반영하지 못하고 있다. 특히, 스트레스는 전통적으로 기초간호학의 주요 탐구 주제이면서 생행동적 간호의 내용과 효과를 증명해 보일 수 있는 대표적인 영역으로 간주되어 왔음에도 불구하고[21] ‘자율신경균형’과 같은 생행동적 개념의 활용이나 병태생리 기전에 기반한 접근이 부족하다[22].

생행동적 모델/연구는 건강 및 건강지표와 관련하여 심리적(psychological), 행동적(behavioral) 및 생물학적(biological) 요소들간 관련성을 탐색하는 과정으로[23], 다차원적 특성을 내포하는 건강문제를 주요 주제로 삼고 있는 간호학 영역에서 특히 강조되고 있다[24]. 생행동적 연구를 위해서는 생행동적 관점을 반영한 개념의 사용과 중재의 개발, 그리고 생행동적 지표를 활용한 효과 측정 노력이 필요하다. 이론적 기틀은 개념적 기틀과 상호교환적으로(interchangeably) 사용되는 용어로[25], 새로운 가설의 개발이나 검증을 통해 학문의 영역을 확장해 나가는데 안내자 역할을 할 뿐만 아니라, 중재 및 치료전략을 개발해 나가는데 일관성을 유지하고 타당성을 확보하는데 중요한 요소가 된다[26,27]. 따라서 생행동적 이론적 기틀의 개발은 과학 학인 간호학의 학문적 정체성을 굳건히 하고 다양한 간호현상에 대한 생행동적 관점의 가설 도출과 검증을 통해 간호학의 영역 확장을 촉진할 수 있다[26].

한편, 스트레스가 직접적으로 자율신경기능의 변화와 함께 다양한 생물학적 및 병태생리학적 기전을 동반함에도 불구하고 스트레스 완화 중재를 위한 생행동적 관점에서의 이론적 기틀에 관한 연구가 여전히 부족함을 알 수 있는데 그 이유 중 하나가 연구현상과 관련된 생행동적 개념이나 병태생리 기전이 익숙하지 않기 때문이다[21]. 이러한 제한점은 스트레스 완화를 위한 효과적인 중재의 개발이나 타당한 중재 결과의 측정을 저해하는 장벽이 될 수 있다[26]. 이에 본 연구에서는 스트레스가 세포노화에 미치는 병태생리 기전을 포괄적으로 고찰하고 이에 기반하여 스트레스 반응 완화를 ‘자율신경균형’ 회복으로 보아 자율신경균형 증진 간호중재가 세포노화에 미치는 효과 연구를 위한 생행동적 관점에서의 이론적 기틀을 구축해 보고자 한다.

### 2. 연구 목적

본 연구의 목적은 통합적인 문헌고찰을 통해 스트레스가 세포노화에 미치는 영향에 대한 기전을 설명하고, 이를 기반으로 자율신경균형 증진 간호중재를 위한 생행동적 관점의 이론적 기틀을 구축해 보고자 하는 것이며, 구체적인 목적은 다음과 같다.

1) 스트레스가 세포노화에 미치는 기전을 고찰한다.

2) 스트레스 반응 완화에 초점을 둔 자율신경균형 증진 간호중재 적용을 위한 생행동적 관점에서의 이론적 기틀을 구축한다.

## 문헌 고찰

### 1. 심리적 스트레스와 세포노화

스트레스 반응은 내·외적 스트레스 요인에 의해 개체와 환경의 상호작용에서 환경의 요구가 개인의 대처자원의 효과를 초과할 때 자기조절의 장애로 나타나는 증상이다[28]. 적절한 스트레스는 긴장감을 유지시켜 업무 수행에 도움을 주지만 과도하게 누적된 만성적 스트레스는 생리적, 심리적 변화를 일으켜 다양한 종류의 신체적 및 정신적 질환을 유발한다[7]. 이처럼 만성적 스트레스가 질병을 일으키는 기전은 스트레스가 체내 여러 병태생리적 변화를 초래하여 세포의 노화를 촉진하기 때문으로 설명하고 있다. 선행 연구에 의하면 장기간의 스트레스는 ‘생체적응 부하(allostatic load)’를 가중시키는데, 이는 반복되거나 만성적 스트레스에 노출된 개인에게 나타나는 신체적 마모와 손상으로 생리적 불균형의 결과로 초래되는 상태로 정의되고 있다[29]. 심리적 스트레스는 신경내분비계와 자율신경계의 변화를 초래하여 세포 및 분자수준에서 복합한 기전들이 서로 피드백을 주고 받으며 상호관련 되는데 이 과정에 관여하는 주요 스트레스 매개물질들은 당질코르티코이드(glucocorticoids), 염증성 사이토카인, 그리고 반응성 활성산소(reactive oxygen species [ROS]) 등이 있다[30]. 스트레스로 인한 HPA 축의 활성화는 혈중 코티졸 분비를 증가시키고 이는 체내 대사율과 미토콘드리아 활성도를 높여 ROS 생성을 촉진시킨다[31]. 전통적으로 당류코티코이드는 항염증 작용을 하는 것으로 알려졌으나 최근의 연구에 따르면 염증성 사이토카인 유전자(pro-inflammatory genes)의 발현을 증가시키는 것으로 보고되고 있다[21,32]. 이처럼 당류코르티코이드는 염증성 사이토카인의 발현 증가 뿐만 아니라 항염증성 사이토카인의 발현을 감소시키는 기전을 통해서도 ROS 생성을 증가시키며[30,32], 염증성 사이토카인 증가도 항산화효소의 활성도를 감소시킨다[33]. 따라서 심리적 스트레스는 자율신경불균형을 초래하여 코티졸 호르몬 및 염증성 사이토카인 분비를 증가시키고 그 결과 ROS 생성 증가 및 항산화효소의 감소로 체내 산화스트레스 수준을 가중시킴을 알 수 있다[10,34,35].

산화스트레스란 ROS의 생성과 분해, 그리고 활성산소로 인한 손상을 복구하는 능력 사이의 불균형 상태를 의미하며[36], 심리적 스트레스와 관련된 호르몬, 염증성 사이토카인 및 대사변화가 세포노화를 초래하는 기전으로 설명되고 있다[37,38]. 산화스트레스의 증가는 특히 텔로미어 DNA 손상과 밀접한 관련이 있는데, 그 이유는 텔로미어 DNA 염기서열은 ROS에 손상받기 쉬운 구조이며[39], 텔로미어를 구성하는 단백질인 쉘터린(shelterin)은 ROS로 인한 DNA 손상을 복구하는 과정을 방해하기 때문이다[40]. 또한 산화된 텔로미어 DNA는 짧아진 텔로미어 길이를 복구시키는 텔로머라제(telomerase)의 활성을 억제하므로 산화스트레스는 텔로미어 길이의 감손(attrition)을 촉진시킨다[41-43].

텔로미어 생물학(telomere biology) 체계는 게놈(genomic)과 세포의 통합성을 유지하는데 핵심적인 역할을 하며 텔로미어와 텔로머라제와 같이 서로 밀접하게 관련된 물질로 구성된다[13]. 텔로미어는 염색체 말단에 위치하는 DNA-단백질 복합체이며, 특정 염기서열(5'-TTAGGG-3')이 반복된 비암호화 영역(non-coding segment)으로, 세포분열시 염색체를 보호하여 염색체의 완전한 복제와 안정성을 유지하는데 매우 중요한 구조이다[44]. 텔로미어는 세포분열시 일어나는 DNA 복제 과정에서 DNA 말단의 일부가 복제되지 못하기 때문에 세포가 한번 분열할 때마다 그 길이가 8~10 base pairs (bp)씩 짧아지게 된다. 그러다가 일정 시점이 되면 짧아진 텔로미어로 인해 더 이상 세포분열하지 못하고 비가역적으로 증식을 멈추게 되면서 손상된 세포가 새로운 세포로 대체되지 못하여 세포노화 상태에 진입하게 된다[13,44]. 세포노화는 조직의 생리적 기능 변화와 위축을 초래하여 신체의 기능적 노화를 가속화시키기 때문에 결국 세포노화가 신체노화를 유발하고 나아가 노화관련질환을 촉발하게 된다[4,12]. 이 과정에서 텔로미어 DNA를 합성할 수 있는 역전사 효소인 텔로머레이스가 작용하여 짧아지는 텔로미어 길이를 복구할 수 있으나[13,30]. 만성적 스트레스는 산화스트레스를 증가시켜 텔로머라제 활성도를 감소시킨다[45]. 현재까지 텔로미어 길이 단축이나 기능장애는 세포노화는 물론, 당뇨병, 심혈관질환, 알츠하이머, 암 등과 같은 노화관련질환과 심리적 디스트레스를 초래할 수 있으며[13,46,47], 나아가 사망 위험률 증가와도 관련이 있는 것으로 알려져 있다[46,48].

한편, 텔로미어의 기능장애는 p53 유전자 발현을 유도하여 미토콘드리아 기능을 조절하는 단백질의 합성을 억제하므로 결국 미토콘드리아 기능장애를 초래할 수 있다[17,49,50]. 미토콘드리아는 인체의 주요 에너지원인 ATP를 생성하는 기능 이외에도 세포주기 조절이나 세포자멸사, 세포분화 등 다양한 생리적 기능을 수행하는 세포내 소기관으로, 이의 기능장애는 여러 노화관련질환과 관련된다[51]. 선행연구를 통해 심리적 스트레스로 인한 자율신경불균형은 미토콘드리아 손상이나 기능장애를 초래하여 노화를 가속화하고 심혈관질환이나 당뇨병 등과 같은 다양한 노화관련질환 위험을 증가시킨다는 사실이 알려져 있다[4,9,12,52,53]. 앞서 언급한 바와 같이 미토콘드리아는 정상적인 대사과정에서 ROS를 생성하기도 하지만 역으로 ROS에 의해 쉽게 손상을 받기도 한다[49,54]. 그러므로 스트레스로 인해 가중된 ROS는 미토콘드리아를 손상시키고, 손상된 미토콘드리아는 기능감소로 더 많은 ROS를 생성시키므로 이로 인해 텔로미어 길이 단축이 가속화될 수 있다는 사실이 알려지면서 텔로미어와 미토콘드리아는 세포노화에 있어 양방향의 상호관련성을 나타낸다는 것이 밝혀졌다[30]. 이로 인해 최근에는 미토콘드리아 역시 세포노화를 반영하는 유용한 지표로 주목을 받고 있는데, 특히 미토콘드리아의 기능을 반영하는 DNA 복제수 (mitochondrial DNA copy number)가 주로 측정되고 있다[17]. 이처럼 스트레스로 인한 세포노화의 과정은 만성적 스트레스 자극이 만성적 심리적 스트레스를 유발하고 이로 인한 생리적 스트레스 반응의 과활성 상태가 지속될 경우 세포노화를 가속화시키는 것으로 요약할 수 있다.

### 2. 심리적 스트레스 완화 중재를 위한 이론적/개념적 기틀 문헌검색

#### 1) 데이터베이스 및 검색어

심리적 스트레스 완화를 위한 비약물적 중재 적용을 위해 선행문헌에서 제안된 이론적/개념적 기틀을 고찰하기 위해 본 연구에서는 국내외 주요 웹 기반 데이터베이스를 중심으로 문헌검색을 실시하였다. 문헌검색 방법 및 과정에 대한 기술은 이론적 기틀 구축을 위한 선행 종설 문헌에서 제시된 방법을 참조하였다[55,56]. 국외문헌은 EMBASE, PubMed 및 CINAHL의 학술연구를 기반으로 문헌을 검색하였고, 국내문헌은 학술연구정보서비스(RISS4u), 한국학술정보(KISS), 과학기술정보통합서비스(NDSL), 및 학술데이터베이스서비스(DBPia)을 활용하여 검색하였다. 국외문헌은 스트레스 완화를 위한 비약물적 중재로서 “mindfulness”, “mindfulness-based stress reduction (MBSR)“, “cognitive behavioral therapy”, “meditation”, “relaxation therapy”, “biofeedback”, “music therapy”, “art therapy”를 주요어로 사용하고, “theoretical framework”, “theoretical model”, “conceptual framework”, “conceptual model”을 조합하여 검색하였다. 국내 문헌 검색을 위해서는 “마음챙김명상”, “인지행동치료”, “인지행동요법”, “명상”, “명상요법”, “명상치료”, “이완요법”, “이완치료”, “이완훈련”, “바이오피드백”, “음악요법”, “음악치료”, “미술요법”, “미술치료”를 주요어로 사용하고, “이론적기틀”, “이론적모델”, “개념적기틀”, “개념적모델”을 조합하여 검색하였다.

#### 2) 문헌선택 과정

**Figure 1.**
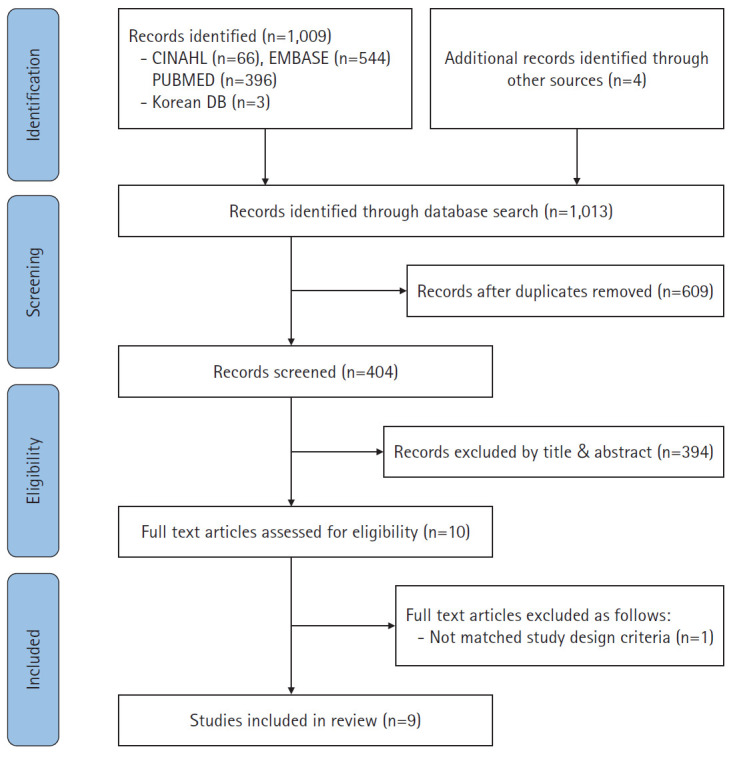
Flow diagram of the study selection process.

본 연구의 문헌검색은 2024년 2월까지 학술지에 게재된 연구논문을 대상으로 시행하였다. 국외 3개의 데이터베이스를 통해 검색된 문헌은 총 1,006편이었고, 국내 4개의 데이터베이스를 통해 검색된 문헌은 총 3편이었다. 검색된 1,013편의 문헌을 대상으로 중복검사를 시행한 결과 609편의 중복문헌이 제거되었다. 이 후 404편의 문헌에 대해 제목과 초록을 확인하여 인간을 대상으로 하지 않은 연구, 이론적 기틀이 제시되지 않은 중재연구 및 기초실험연구 394편이 제외되었고, 이 후 2명의 연구자가 독립적으로 전문을 검토하여 그 결과를 합의하였다. 그 결과 총 9편의 문헌이 검색되었고, 간호학 분야 논문은 1편이 포함되었다(Figure 1).

### 3. 심리적 스트레스 완화 중재를 위한 이론적/개념적 기틀 분석

스트레스는 다양한 병태생리적 기전을 통해 건강상태와 질병발생에 영향을 미칠 수 있기 때문에 스트레스 자극으로 인한 스트레스 반응을 완화시키기 위한 다양한 중재가 소개되어 왔다. 스트레스 완화를 위해 소개된 대표적인 중재에는 마음챙김(mindfulness) 혹은 마음챙김기반 스트레스 완화 중재(MBSR), 명상(meditation), 인지행동요법(cognitive behavioral therapy), 음악요법(music therapy) 등이 보고되었다. 이들 중재들은 전통적으로 개인의 정서적 반응성을 감소시키고 인지적 평가를 강화하여 자신과 스트레스를 유발하는 사고 및 사건에 대한 인식을 변화시키거나[57,58] 스트레스 지각과 스트레스 각성을 감소시켜[34,59]. 걱정이나 불안 같은 부정적인 감정을 완화시키고 긍정적인 감정을 높여준다[60,61]. 그 결과, 생리적 각성과 코티졸, 심박동 수 및 혈압이 감소되고[62,63] 심혈관 지표가 개선되는 것은 잘 알려져 있으며[64,65]. 이러한 스트레스 완화 중재의 효과가 세포수준이나 유전자 발현수준에까지 영향을 미칠 수 있다는 근거가 최근 보고되면서[66,67] 심리적 스트레스가 질병을 초래하는 기전에 기반을 둔 기틀이나 모델을 구축하려는 노력이 일부 시도되고 있음을 알 수 있었다.

전체적으로 보았을 때 스트레스 완화를 위한 비약물적 중재연구는 다양한 분야에서 다양한 중재방법을 적용하여 효과를 검증한 것으로 분석되었으나 이론적 기틀을 구축하여 이를 중재연구에 실제 적용한 문헌은 검색되지 않았다. 대신 Table 1에 제시된 바와 같이 본 연구에서 검색된 문헌은 모두 이론적 기틀 구축에 국한된 종설연구임을 알 수 있었으며, 이중 1편은 체계적 문헌고찰 방법을 사용하였다[68]. 본 연구에서 최종적으로 검색된 9편의 문헌 중 기존에 알려진 이론(모이론, mother theory)을 기반으로 이론적 기틀을 도출한 논문이 2편이었고[69,70], 문헌고찰 내용을 기반으로 이론적 기틀을 구축한 문헌이 7편이었다[55,56,66-68,71,72]. 주목할 부분은 7편의 종설 연구 중 2편의 문헌에서 생물학적 관점에서의 이론적 기틀을 제시하였다는 점이다. Black 등[66]은 마음챙김 명상 중재를 위해 사회 유전학(social genomics)적 문헌을 기반으로 가설적 기틀을 제시하였고, Conklin 등[67]은 명상과 관련된 텔로미어 생물학 문헌으로부터 이론적 모델을 구축함으로써 스트레스 완화 중재를 위한 이론적 기틀로서 생물학적 혹은 생행동적 관점에서의 접근이 시도되고 있음을 알 수 있었다.

한편, 9편의 문헌에서 제시하고 있는, 혹은 기술하고 있는 측정지표 및 결과 지표는 사회심리적 지표만을 포함한 문헌이 4편[55,56,69,70], 생물표지자(biomarker)만을 포함한 문헌이 3편[66,67,71], 그리고 사회심리적 지표와 생물표지자를 모두 포함한 문헌이 2편이었다[68,72]. 스트레스 완화 중재 효과 측정을 위한 생물표지자에는 스트레스 관련 생리적 지표(코티졸, 카테콜아민, 심박변이도, 피부전도, 활력징후 등), 심리신경면역지표(심리적·생리적·신경계·내분비계·면역계 반응), 유전자 발현지표, 텔로미어 길이 및 텔로머라제 활성도 등을 포함하였다. 이는 최근들어 스트레스와 관련된 새로운 분자생물학적 근거들이 밝혀지면서 전통적인 심리 및 생리적 지표 이외 일부 문헌이지만 유전자 발현과 세포노화 지표인 텔로미어까지 다루고 있음을 알 수 있었다. 본 연구에서 분석한 9편의 문헌 중 간호학 분야 문헌은 국내 및 국외 포함 2편으로, 1편은 심리적 지표를[70] 다른 한편은 생리적 지표 측정을 제안하였다[71].

## 연구결과 및 논의

### 1. 자율신경균형 증진 간호중재를 위한 세포노화 기전 기반 이론적 기틀 구축

**Figure 2.**
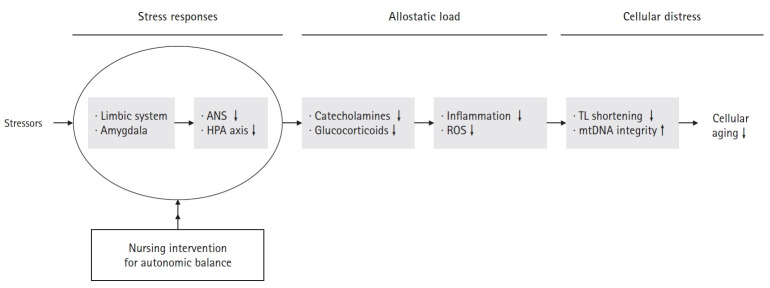
A biobehavioral theoretical framework for nursing interventions to promote autonomic balance. ANS = autonomic nerve system; HPA = hypothalamic-pituitary-adrenal; ROS = reactive oxygen species; TL = telomere length; mtDNA = mitochondrial DNA.

심리적 스트레스와 자율신경기능은 마음과 몸(mind-body)이 통합되는 대표적인 생리적 현상 중 하나로, 심리적 스트레스는 자율신경기능의 변화를 초래한다. 따라서 만성적인 스트레스는 자율신경계의 과도한 활성화로 인해 불균형을 초래하고 이러한 상태가 장기간 지속될 경우에는 다양한 병태생리 기전을 통해 세포노화를 초래하게 된다[13,30,34,73]. 세포노화는 신체의 노화, 혹은 노화관련질환의 발생 기전으로 알려지면서[4,12,52,53] 심리적 스트레스와 세포노화의 병태생리적 경로에 대한 관심이 간호학 분야에서도 나타나기 시작하였고[22,74-76] 이러한 기전을 간호학적 지식으로 통합해 가기 위해서는 간호학적 관점에서의 접근이 필요하다[77]. 이 과정에서 이론적 기틀은 새로운 가설의 개발이나 검증을 위한 안내자 역할을 할 뿐만 아니라, 중재전략을 개발해 나가는데 일관성을 유지하고 타당성을 확보하는데 유용한 도구가 될 수 있다[26,27]. 이에 본 연구에서는 문헌에서 보고된 병태생리 기전으로부터 귀납적인 방법을 이용하여 간호학적 측면에서 접근할 수 있는 자율신경균형 스트레스 반응 완화중재를 위한 생행동적 관점에서의 이론적 기틀을 Figure 2와 같이 제시하였다.

본 연구에서 제안하고 있는 생행동적 모델의 이론적 기틀은 지금까지 스트레스 완화 중재로 불리워 왔던 다양한 간호중재를 생행동적 모델과 통합해 보고자 시도된 것으로 이를 위해 스트레스로 인해 세포노화가 초래되는 병태생리적 기전의 주요내용을 6개의 진술로 도출하여 도식화하였다. 그 내용을 요약하면 순차적으로 다음과 같다. 첫째, 스트레스 자극은 뇌의 변연계와 편도체를 활성화시켜 생리적 스트레스 반응을 유발한다[62,78]. 둘째, 스트레스 반응은 생리적으로 교감신경계와 HPA 축을 활성화시키고 카테콜아민과 글루코코르티코이드의 분비를 증가시킨다[29]. 셋째,만성적 생리적 스트레스 반응은 생체적응 부하를 증가시켜 자율신경불균형을 초래하고 염증성 사이토카인의 분비를 증가시킨다[30,32]. 넷째, 염증성 사이토카인의 분비는 ROS 생성증가 및 항산화기전의 감소를 초래한다[33]. 다섯째, ROS의 증가로 인한 산화스트레스는 텔로미어 길이를 단축시키고 미토콘드리아 DNA 복제수를 감소시킨다[18,30]. 여섯째, 텔로미어 길이 단축과 미토콘드리아 복제수 감소는 결과적으로 세포노화를 촉진시킨다[13,30,34,73]. 이처럼 심리적 스트레스가 세포노화를 유발시키는 기전은 스트레스를 완화시키는 MBSR이나 명상과 같은 중재를 통해 텔로미어 길이 단축 속도를 늦추거나[79] 텔로머라제 활성도 증가를 통해[80] 세포노화를 억제할 수 있음을 일부 연구를 통해서도 확인된 바 있다[34]. 따라서 스트레스 완화 간호중재는 자율신경균형을 회복하도록 도와 스트레스 반응으로 촉발되는 일련의 세포노화 기전을 차단하거나 완화시켜줄 수 있을 것으로 가정해 볼 수 있으며, 자율신경기능균형 증진을 위한 간호중재 계획시 본 연구에서 제안하는 세포노화 기전 기반 생행동적 이론적 기틀이 새로운 간호학적 근거의 생성과 활용을 위한 대안이 될 수 있을 것이다.

### 2. 생행동적 관점에서의 이론적 기틀의 활용

이론적 기틀은 특정 현상을 설명하기 위하여 관련된 개념과 그 개념들 간의 관계를 논리적으로 표현한 것으로, 현상을 통합적으로 이해할 수 있도록 해 줄 뿐만 아니라 연구를 위한 다양한 가설을 유도하도록 안내하고 나아가 보다 효과적인 중재 프로그램의 개발을 촉진한다[26]. 그러나 이론적 기틀의 부재나 모호함은 중재의 이론적 근거를 설명하지 못하고 중재내용의 구성이나 효과측정의 타당성도 확보하기 어렵기 때문에 연구와 실무를 안내하는데 제한이 있다[81]. 특히 마음과 몸의 상호작용을 반영하는 건강문제 탐구시 현상에 대한 기전을 충분히 이해하고 이를 이론적 기틀내에 통합할 수 있어야 한다[26].

지금까지 스트레스 완화 중재연구에 적용된 이론적/개념적 기틀은 대체로 스트레스 대처 이론[82]에 기반을 두어 개발되었으나 이 모델은 스트레스 반응 자체가 생리적 반응을 동반함에도 불구하고 모델내 이러한 기전들이 충분히 반영되지 못한 제한점이 있어 왔다[21]. 이로 인해 생행동적 관점의 간호중재의 효과가 세포수준까지 측정되지 못하거나 간과되어 왔을 가능성이 높다. 이러한 측면에서 본 연구에서 제안하고 있는 이론적 기틀은 스트레스가 심리적, 정서적 문제에 그치지 않고 세포손상, 나아가 세포노화 및 노화관련질환에까지 이를 수 있음을 간호학적 관점으로 통합함으로써 생행동적 간호연구, 간호이론 및 간호실무에 유용하게 활용될 수 있을 것이다.

전통적으로 자율신경기능은 생리학적 현상이자 용어이며 자율신경불균형이 질병의 병태생리적 기전에 미치는 기전은 의학적 관점에서 주로 설명되어 왔는데 이는 결과론적이고 교정할 수 없는 기전으로 간주되어 왔다. 그러나 최근들어, 자율신경기능은 가변성이 있고 교정가능한 체계라는 주장이 설득력을 얻고 있다[83,84]. 일부 보고에 따르면 신체활동, 영양, 수면 등과 같은 생활습관을 개선시키면 자율신경균형을 증진시킬 수 있으며[19,20], 그 결과 질병상태의 개선은 물론 유전물질인 텔로미어 길이의 연장까지도 가능한 것으로 알려져 있다[8,16,85]. 따라서 자율신경균형 증진을 위한 간호중재 계획시 본 연구에서 제안하는 세포노화 기전 기반 생행동적 이론적 기틀의 활용은 다양한 기초간호학적 가설의 유도를 촉진하고 중재효과의 타당성과 일관성을 확보하는데 유용할 것으로 생각된다. 이론적 기틀로부터 유도된 가설은 경험적으로 검증이 가능하므로[66] 이를 통해 세포노화의 병태생리적 기전을 간호학적 지식의 영역으로 통합하고 과학적인 근거를 축적해 나가도록 촉진할 것이다.

한편, 스트레스는 생행동적 간호연구의 주요 주제가 되어 온 만큼 다른 간호학적 개념보다는 생물표지자를 많이 활용하고자 노력해 왔다. 그중 신경내분비 지표인 코티졸이 가장 많이 측정되었고, 그 다음으로 카테콜아민으로 에피네프린, 노르에피네프린 및 도파민이 측정되었으며, 그 외 자율신경계 지표인 혈압, 맥박, 심박변이도[74,86], 그리고 염증지표인 사이토카인과 C-반응 단백질 등도 소개되었다[21]. 이러한 지표들은 스트레스 관련 간호의 효과를 보여주는데 유용하게 활용되어 왔으나[74] 세포수준의 변화를 반영하는 정도에는 미치지 못하는 것으로 생각된다. 또한 인접하고 있는 여러 학문 분야에서 다양한 분자생물학적 측정기법을 활용하고 있는 것에 비해 간호학 분야에서의 활용은 미흡한 것으로 보인다. 이러한 추세는 학문간의 경계가 허물어지고 다학제간 연구가 점차 활발해져가고 있는 시점에서 간호학의 참여와 기여를 제한하는 요소가 될 수 있다. 따라서 스트레스 반응 완화를 위한 자율신경균형 증진 간호중재 개발시 생행동적 관점의 이론적 기틀을 적용함으로써 간호중재의 효과측정 지표로 다양한 세포노화 생물표지자의 활용을 촉진할 수 있을 것이며, 이를 통해 과학이자 예술인 간호의 근본적인 철학에 다가갈 수 있을 것이다[21].

본 연구를 통해 스트레스로 인해 유발되는 세포노화의 기전을 간호학에서 접근이 가능한 생행동적 관점에서 탐색하고 스트레스 완화 중재를 자율신경균형의 관점으로 통합한 생행동적 간호중재의 이론적 기틀을 제시하였다는 점에서 의의를 찾아볼 수 있다. 이는 스트레스-세포노화 기전에 개입할 수 있는 간호중재와 관련된 다양한 가설유도를 촉진하고 새로운 생리적 지표의 발굴을 촉진할 수 있을 것이다. 다만, 본 연구에서 제시하는 생행동적 이론적 기틀은 세포노화의 기전을 스트레스의 관점에서만 탐색하였으며, 스트레스가 세포노화에 미치는 다양한 기전을 모두 포함하지 못하였으므로 추후연구를 통해 이론적 기틀을 확장하고 정련화하는 과정이 필요하다.

## 결론 및 제언

본 연구는 스트레스가 세포노화를 유발하는 병태생리적 기전을 설명하고, 여기에 근거하여 스트레스 반응 완화를 위한 자율신경균형 증진 간호중재시 적용할 수 있는 생행동적 관점의 이론적 기틀을 제안하고자 시도된 종설연구이다. 연구결과, 스트레스로 인해 유발되는 스트레스 반응은 카테콜아민과 글루코코르티코이드 분비를 증가시켜 생체적응 부하를 가중시키고 그 결과 염증반응과 산화스트레스를 유발하여 텔로미어 길이 단축과 미토콘드리아 DNA를 손상시키고 이로 인해 세포노화가 초래될 수 있음을 확인할 수 있었다. 이러한 기전에 근거하여, 스트레스 반응 완화를 위한 자율신경균형을 증진시킴으로써 세포노화로 진행되는 병태생리적 과정을 지연시키거나 억제시킬 수 있을 것으로 보아 생행동적 관점의 이론적 기틀을 구축하였다. 따라서 본 연구에서 제시하고 있는 생행동적 이론적 기틀은 스트레스가 세포노화를 초래하는 기전에 기반을 둠으로써 스트레스의 생행동적 측면에 대한 이해를 확장하고 가설유도를 촉진하며 스트레스 완화 간호중재의 타당성과 효과성을 가시화하는데 도움이 될 것이다. 본 연구결과를 바탕으로 한 추후연구를 위해 다음과 같이 제언하고자 한다.

첫째, 본 연구에서 제시하고 있는 생행동적 이론적 기틀을 활용하여 스트레스 반응 완화를 위한 자율신경균형 증진 간호중재 개발연구를 제안한다.

둘째, 본 연구에서 제시하고 있는 이론적 기틀로부터 다양한 가설을 도출하여 이를 검증하는 연구가 필요하다.

셋째, 스트레스가 세포노화를 유발하는 다양한 기전을 포함한 통합적이고 다차원적인 이론적 기틀 구축 연구를 제언한다.

## Figures and Tables

**Table 1.** Summary of the Theoretical Framework of Non-pharmacological Interventions for Perceived Stress Reduction

Author (yr)	Research design	Intervention	Theoretical background/Evidence source	Measures/Outcomes
Khoury et al. (2023)	Methodological study	Interpersonal mindfulness (MBSR)	⋅S-ART	⋅Emotional & mindfulness states
			- Self-awareness	⋅Interpersonal interactions
			- Self-regulation	
			- Self-transcendence	
Prather et al. (2022)^†^	Review	Mindfulness-based intervention	⋅Selye’s stress response theory	⋅Function
			⋅McEwen’s allostatic overload	- Emotional
				- Physical
				- Social
				- Cognitive
				⋅Quality of life
Black et al. (2019)	Review	Mindfulness meditation	⋅Social genomics literature	⋅Gene expression profiles
Conklin et al. (2019)	Review	Meditation training	⋅Literature regarding telomere biology in relation to meditation	⋅Telomere outcomes
				- Telomere length
				- Telomerase activity
Santiago & Colussi (2018)	Review	MBSR	⋅Literature	⋅Feasibility
Landis-Shack et al. (2017)	Narrative review	Music therapy	⋅Literature	⋅Increased pleasures
				⋅Group improvisation
				⋅Reduction of stress
				⋅Cathartic release
Kim & Hon (2015)^†^	Review	Cognitive behavioral therapy	⋅Literature	⋅Physiological indicators
Fancourt et al. (2014)	Systematic review	Music	⋅Literature	⋅ Psychoneuroimmunological effects
				- Psychological
				- Physiological
				- Neurological
				- Endocrinological
				- Immunological
Gard et al. (2014)	Review	Yoga	⋅Literature	⋅Cognitive, emotional & behavioral output
				⋅Autonomic output

MBSR = Mindfulness-based stress reduction.

^†^Published in the nursing field.

## Data Availability

Please contact the corresponding author for data availability.
